# Inhibition of Human Dendritic Cell ER Stress Response Reduces T Cell Alloreactivity Yet Spares Donor Anti-tumor Immunity

**DOI:** 10.3389/fimmu.2018.02887

**Published:** 2018-12-06

**Authors:** Brian C. Betts, Frederick L. Locke, Elizabeth M. Sagatys, Joseph Pidala, Kelly Walton, Meghan Menges, Jordan Reff, Asim Saha, Julie Y. Djeu, John V. Kiluk, Marie C. Lee, Jongphil Kim, Chang Won Kang, Chih-Hang Anthony Tang, Jeremy Frieling, Conor C. Lynch, Alan List, Paulo C. Rodriguez, Bruce R. Blazar, Jose R. Conejo-Garcia, Juan R. Del Valle, Chih-Chi Andrew Hu, Claudio Anasetti

**Affiliations:** ^1^Department of Blood and Marrow Transplantation and Cellular Immunotherapy, Tampa, FL, United States; ^2^Department of Immunology, Moffitt Cancer Center, Tampa, FL, United States; ^3^Division of Hematology, Oncology, and Transplantation, University of Minnesota, Minneapolis, MN, United States; ^4^Department of Hematopathology and Laboratory Medicine, Moffitt Cancer Center, Tampa, FL, United States; ^5^Division of Blood and Marrow Transplantation, Department of Pediatrics, Masonic Cancer Center, University of Minnesota, Minneapolis, MN, United States; ^6^The Center for Immunology, University of Minnesota Medical School, Minneapolis, MN, United States; ^7^Comprehensive Breast Program, Moffitt Cancer Center, Tampa, FL, United States; ^8^Department of Biostatistics and Bioinformatics, Moffitt Cancer Center, Tampa, FL, United States; ^9^Department of Chemistry, University of South Florida, Tampa, FL, United States; ^10^Department of Translational Tumor Immunology, The Wistar Institute, Philadelphia, PA, United States; ^11^Department of Tumor Biology, Moffitt Cancer Center, Tampa, FL, United States; ^12^Department of Malignant Hematology, Moffitt Cancer Center, Tampa, FL, United States

**Keywords:** GvHD, GvL, er stress, XBP-1S, dendritic cell (DC)

## Abstract

Acute graft- vs. -host disease (GVHD) is an important cause of morbidity and death after allogeneic hematopoietic cell transplantation (HCT). We identify a new approach to prevent GVHD that impairs monocyte-derived dendritic cell (moDC) alloactivation of T cells, yet preserves graft- vs.-leukemia (GVL). Exceeding endoplasmic reticulum (ER) capacity results in a spliced form of X-box binding protein-1 (XBP-1s). XBP-1s mediates ER stress and inflammatory responses. We demonstrate that siRNA targeting XBP-1 in moDCs abrogates their stimulation of allogeneic T cells. B-I09, an inositol-requiring enzyme-1α (IRE1α) inhibitor that prevents XBP-1 splicing, reduces human moDC migration, allo-stimulatory potency, and curtails moDC IL-1β, TGFβ, and p40 cytokines, suppressing Th1 and Th17 cell priming. B-I09-treated moDCs reduce responder T cell activation via calcium flux without interfering with regulatory T cell (Treg) function or GVL effects by cytotoxic T lymphocytes (CTL) and NK cells. In a human T cell mediated xenogeneic GVHD model, B-I09 inhibition of XBP-1s reduced target-organ damage and pathogenic Th1 and Th17 cells without impacting donor Tregs or anti-tumor CTL. DC XBP-1s inhibition provides an innovative strategy to prevent GVHD and retain GVL.

## Introduction

DCs are a relevant biologic target for GVHD prevention (1), though current immune suppressive GVHD treatment strategies primarily act upon donor T cells (2–4). Beyond antigen presentation and costimulation, DCs produce proinflammatory cytokines that fuel alloreactive T cells (5). Activated DCs secrete IL-1β via the inflammasome (6), which promotes the differentiation of pathogenic Th17 cells (7, 8). In response to ER stress, the inflammasome is regulated by XBP-1, a transcription factor that is spliced and activated by the RNAse subunit of IRE1α (9, 10). XBP-1s allows the ER to synthesize lipids, expand its size, and produce chaperones to fold ER client proteins that can protect stressed cells from death (9, 11, 12). Prolonged ER stress leads to hyperactivation of IRE1α and secondary inflammasome activation via interactions between XBP-1s and NLRP3 (9, 13). Additionally, XBP-1s regulates DC responses to inflammatory stimuli, such as lipopolysaccharide (LPS) (14).

Inflammasome activity within recipient tissue directly impacts murine GVHD severity (15). NLRP3-deficient mice are partially protected from acute GVHD, while transfer of allogeneic NLRP3-deficient donor T cells to normal hosts has no protective effect (15). This suggests that inflammasome activity in antigen present cells, and/or DCs, contribute to acute GVHD. Furthermore, microRNA-155 is known to regulate DC inflammasome activity (15). MicroRNA-155 deficient murine DCs exhibit impaired IL-1β production from the inflammasome, and such recipient mice also develop significantly less severe acute GVHD (16). In other studies, GVHD but not no GVHD transplant control mice were shown to induce inflammasome activation in myeloid-derived suppressor cells within the first 5 days post-transplant resulting in a loss of their suppressor function (17). Inflammasome activation has been linked to the expression of the purinergic receptors P2x7R and P2Y2, that can sense ATP from damage-associated molecular patterns produced during GVHD, while neutralizing ATP or purinergic receptor triggering reduces GVHD (18, 19). Similarly in patients, high ATP levels have been documented in the peritoneal fluid of patients with severe GVHD (18). Altogether, this evidence strongly supports the pathogenic role of the inflammasome in acute GVHD biology (20).

However, translating this concept to clinical transplantation remains a challenge. Administering IL-1 receptor antagonist failed to prevent acute GVHD in some (21), but not all (22) murine models, and in patients treated in the early post- or peri-HCT period, respectively (23). While the inflammasome and IL-1β are biologically relevant to GVHD pathogenesis, the best strategy to target IL-1β and control donor T cells remains uncertain (15, 23).

Given the fundamental contributions of the inflammasome to acute GVHD and XBP-1s as a regulator of inflammasome activation, targeting ER stress via XBP-1s inactivation could represent a novel strategy to prevent acute GVHD. In support of this possibility is work in autoimmune syndromes showing that blocking ER stress with tauroursodeoxycholic acid ameliorates experimental autoimmune encephalomyelitis and reduces Th17 differentiation (24). In a donor *B cell* dependent *chronic* GVHD model, suppressing XBP-1s in donor B cells reduces murine chronic GVHD (25). While these findings in murine chronic GVHD are important, translational questions regarding how the ER stress response influences human acute GVHD pathogenesis were not addressed.

Our present work is distinct from observations in murine chronic GVHD, as we demonstrate that siRNA knock down or a small molecule inhibitor of XBP-1s can ameliorate DC-allostimulation of human T cells, and using a human skin xenograft model we show that pharmacologic inhibition of XBP-1s can reduce donor alloreactivity *in vivo*. Mechanistically, we also demonstrate how blocking the ER stress response of DCs impacts responding donor T cell activation and differentiation. Herein, we provide human data that support XBP-1s^+^ DCs are relevant biologic targets to prevent acute GVHD, without loss of Treg function or anti-tumor activity by CTLs and NK cells.

## Materials and Methods

### Medium

Unless otherwise stated, cells were cultured in complete RPMI supplemented with 10% heat-inactivated, pooled human serum (26).

### mAbs and Flow Cytometry

Fluorochrome-conjugated anti-mouse or -human monoclonal antibodies included anti-CD3, CD4, CD8, CD25, CD25RO, CD83, CD86, HLA-DR, CD127, CCR6, CCR7, Ki-67, Foxp3, IFNγ, IL-4, IL-17A, XBP-1s, and phosphorylated STAT3 Y705 (Supplemental Table 1). Live/Dead Fixable Yellow Dead Cell Stain (Life Technologies) was used to determine viability. Live events were acquired on a FACSCanto (FlowJo software, ver. 7.6.4).

### moDC Phenotyping

LPS-mature moDCs were surfaced stained for CD83, followed by fixation, permeabilization (eBioscience) and intracellular staining for XBP-1s. All other moDC maturation phenotyping was performed as described (19, 21).

### siRNA Knock Down of XBP-1

Immature, human moDCs were loaded with XBP-1 or control siRNAs (Dharmacon) using polyethyleneimine (Polyplus), and then stimulated with LPS (1 μg/ml) for 24 h in the presence of B-I09 (20 μM) or DMSO (0.1%). XBP-1 knock down was confirmed by flow cytometry. The moDCs were then used as stimulators in 5-day alloMLRs.

### moDC Experiments and alloMLRs

Immature human moDCs were cytokine-generated and differentiated with LPS as described (27). B-I09, XBP-1s inhibitor, was synthesized and purified as reported (28). For XBP-1s expression experiments, moDCs were stimulated with LPS for 24 h while treated with B-I09 (20 μM) or DMSO (0.1%). Supernatant cytokines were quantified using commercial ELISA kits (Thermo Fisher Scientific Inc) after 24 h of LPS-stimulation in the presence of B-I09 or DMSO. RT-PCR to analyze the levels of regulated IRE1-dependent decay (RIDD) substrates, and total and spliced XBP-1 mRNA was performed after LPS stimulation.

moDC chemotaxis was quantified by migration through a 5 μm pore filter in the chamber of a 24-well transwell plate (29). The lower chamber was filled with 500 μl RPMI enriched with 10% heat-inactivated, human pooled serum and B-I09 (20 μM) or DMSO (0.1%). moDCs pre-treated with B-I09 or DMSO during LPS-maturation were added to the upper chamber at 1 × 10^5^/50 μl. CCL19 or CCL21 (300 ng/ml, R&D systems) was added to the lower well and moDC migration was analyzed after 3 h.

moDC stimulatory capacity was measured in 5-day allogeneic mixed leukocyte reactions (alloMLR). Purified T cells were obtained from healthy human donors (OneBlood) as described (26). AlloMLRs were plated at a moDC:T cell ratio of 1:30. The MLRs consisted of DMSO (0.1%) or B-I09 (20 μM) pre-treated moDCs, DMSO or B-I09 treated MLR medium only, or both. T cell proliferation was determined by a colorimetric assay (Promega) (26, 30).

### Calcium Flux Assay

Human moDCs were stimulated with LPS (1 μg/ml) for 24 h in the presence of B-I09 (20 μM) or DMSO (0.1%), and then used to stimulate allogeneic T cells in 5-day alloMLRs. The T cells were then rested for 24 h at 37°C after primary stimulation, transferred to FluoroDish (WPI, 35 mm, 5 × 10^5^ T cells/200 μl) plates coated with Cell-Tak (Corning), loaded with Fluo-4 dye (Thermofisher) for 30 min and washed, and finally restimulated with fresh B-I09- or DMSO-treated moDCs (3 × 10^4^) during live cell imaging to monitor calcium flux in real time (Moffitt Cancer Center, Analytic Microscopy Core).

Live T cells were observed with a Leica TCS SP8 AOBS laser scanning confocal microscope through a 20X/0.8NA or 40X/1.3NA Plan Apochromat objective lens (Leica Microsystems CMS GmbH, Germany). A 488 nm laser line was applied to excite the sample and tunable emission was set to capture the Fluo-4 spectrum. Images were captured at 400 Hz scan speed with photomultiplier detectors and LAS X software version 3.1.5 (Leica Microsystems).

Time lapse images were analyzed using the Definiens Tissue Studio v4.7 (Definiens AG, Munich, Germany) software suite. The green fluorescent channel images were segmented by green intensity and cell size. The image was analyzed as an 8 bit image and intensity was measured from 0 to 255 grayscale fluorescent units. The cells were then quantified for green intensity per field for each time point imaged and then plotted for intensity over time.

## Reverse Transcription and Quantitative Polymerase Chain Reaction (qPCR) to Detect the Expression Levels of RIDD Substrates

Human moDCs were stimulated with LPS (1 μg/ml) for 24 h in the presence of B-I09 (20 μM) or DMSO (0.1%). moDCs were then harvested and total RNA was isolated using TRIzol reagent (Invitrogen). Complementary DNA was synthesized from RNA using Maxima H Minus reverse transcriptase (Thermo Scientific). The following sets of primers were used together with iTaq Universal SYBR Green Supermix (Roche) in qPCR to detect the expression levels of human GAPDH (GGA TGA TGT TCT GGA GAG CC and CAT CAC CAT CTT CCA GGA GC); human XBP1s (CTG AGT CCG AAT CAG GTG CAG and ATC CAT GGG GAG ATG TTC TGG); human XBP1t (TGG CCG GGT CTG CTG AGT CCG and ATC CAT GGG GAG ATG TTC TGG); human Bloc1S1 (CCC AAT TTG CCA AGC AGA CA and CAT CCC CAA TTT CCT TGA GTG C); human CD59 (TGA TGC GTG TCT CAT TAC CAA AGC and ACA CAG GTC CTT CTT GCA GCA G); and human Scara3 (AAC TTC CTG CAC ACA CTG GC and CAA ACC AGT TGC ACA TCC AG).

### Treg Experiments

Tregs were defined as CD4^+^, CD127^−^, CD25^+^, Foxp3^+^ cells (31, 32). Treg potency was determined using suppression assays (26). DMSO (0.1%) or B-I09 (20 μM) was only added to the initial culture to expand the Tregs. No drug was added to the suppression assay medium. Conventional, alloreactive T cell (Tconv) proliferation was measured by Ki-67 expression using flow cytometry. To test the effect of XBP-1s blockade on natural (nTreg) or *in vitro* induced Tregs (iTreg), circulating Tregs were isolated from healthy donor blood by magnetic bead purification (CD4^+^, CD25^+^). Tconv (CD4^+^, CD25^−^) were also purified from the donor sample and stimulated with allogeneic moDCs and IL-2 for iTreg differentiation. The enriched nTregs were also cultured with IL-2 (20IU/ml) and allogeneic moDCs (pretreated with DMSO or B-I09) at a ratio of 1:30. DMSO (0.1%) or B-I09 (20 μM) was added to the co-culture once on day 0 as indicated. After 5 days, the cells were harvested and analyzed by flow cytometry. Tregs were enumerated using CountBright beads (Thermo Fisher Scientific Inc). In select experiments, TGFβ1 (4 ng/ml) (R&D Systems) was added to the medium on alternating days.

### Th1, Th2, and Th17 Phenotype Experiments

T cells were cultured with DMSO- or B-I09-pretreated, allogeneic moDCs, DMSO (0.1%) or B-I09 (20 μM) was added once on day 0. For Th17 experiments only, the T cells were first CD4-purified by magnetic bead isolation and supplemented with IL-1β or TGFβ as indicated, and anti-IFNγ antibody (26). On day +5, the T cells were harvested and stained to identify the following T helper subsets: Th17 - CD4^+^, IL-17A^+^; Th1 - CD4^+^, IFNγ^+^; and Th2 - CD4^+^, IL-4^+^.

### Tumor Lysis Experiments and T Cell Recall Response

Human peripheral blood mononuclear cells (PBMCs, 5x10^5^) were stimulated with irradiated (30Gy) U937 cells (American Type Culture Collection) at a 1:1 ratio on day 0 and +7. DMSO (0.1%) or B-I09 (20 μM) was added on day 0. CD8^+^ T cells were isolated on days +12-14 (to prevent non-specific killing by NK cells), and then cultured with fresh U937 cells at the stated effector-to-target ratios for 4 h at 37°C (26). Unprimed CD8^+^ T cells served as a negative control. No drug was added. Tumor lysis was determined by a colorimetric LDH release assay (Thermo Fisher Scientific Inc) (26, 33). Percent lysis was calculated as follows: [(test optical density (OD) – spontaneous OD)/(maximum OD – spontaneous OD)] × 100 (26, 33).

To determine T cell recall response to nominal antigen, T cells were cultured with autologous moDCs loaded with a mixed CMV, EBV, influenza, and tetanus peptide pool (JPT). DMSO (0.1%) or B-I09 (20 μM) was added once on day 0 of the culture. T cell proliferation was determined after 3 days of culture (34).

### NK Cell Experiments

Human natural killer cells (NK cells) were isolated from healthy donor PBMCs by magnetic bead purification (Miltenyi Biotec Inc). NK cells were cultured with K562 cells at the stated effector-to-target ratios for 5 h at 37°C in the presence of DMSO (0.1%) or B-I09 (20μM) (35). Tumor lysis was determined by a colorimetric LDH release assay (33, 35).

NK cell proliferation was assessed by allogeneic moDC (moDC: NK cell ratio 1:10) or cytokine stimulation (IL-2 200 IU/ml and IL-15 10 ng/ml) (35). DMSO (0.1%) or B-I09 (20 μM) was added once on day 0 of the culture. NK cell proliferation was determined after 5 days using a colorimetric assay.

### Xenograft Model and *in vivo* CTL Generation

NSG mice were transplanted with a 1 cm^2^ human skin graft using a well-established model (33, 36, 37). Skin was procured from consented mastectomy patients (MCC 17634, an IRB-approved protocol). After 30 days of rest, mice received 5 × 10^6^ fresh, human PBMCs (OneBlood) i.p. using a random donor allogeneic to the skin graft (26, 36, 37). Each transplant experiment used a unique donor pair of skin and PBMCs. B-I09 30 mg/kg or a polyethylene glycol-based vehicle (26) was given by i.p. injection 5 days a week for 3 weeks. On day +21, mice were humanely euthanized; skin grafts, host lung, host liver, and host spleen were harvested for analysis. Skin rejection and xenogeneic GVHD scoring was performed blinded according to standard criteria (26, 33, 38). Processed spleens cells were phenotyped by flow cytometry. To generate CD8^+^ CTL *in vivo*, mice were transplanted with 30 × 10^6^ human PBMCs and also received irradiated U937 cells (10x10^6^) on day 0 and +7 (26, 33). Control mice received PBMCs alone without tumor. Mice did not receive skin grafts for these experiments. Mice received B-I09 or vehicle exactly as stated. On days +10-12, the mice were humanely euthanized and the spleens were harvested. Human CD8^+^ T cells within the spleens were purified by magnetic beads. Tumor lysis assays were performed *in vitro*.

### NSG Mice for Xenograft Model and *in vivo* CTL Generation

NSG mice (male or female, 6–24 weeks old) were used in the described *in vivo* experiments. NSG mice were purchased from The Jackson Laboratory and raised at the Moffitt Cancer Center vivarium. Experiments were performed according to an Institutional Animal Care and Use Committee (IACUC)–approved protocol in adherence to the National Institutes of Health's Guide for the Care and Use of Laboratory Animals.

### Statistical Analysis

Data are reported as mean values ±SEM. Normality was tested by the Anderson-Darling test. For comparisons of dependent data, the paired *t*-test was used. The Mann-Whitney test was used for comparisons of *in vivo* mouse and patient skin sample data. ANOVA was used for group comparisons, including a Dunnett's or Tukey post-test with correction for multiple-comparisons. The statistical analysis was conducted using Prism software version 5.04 (GraphPad). Statistical significance was defined by *P* < 0.05 (two-tailed).

## Results

### XBP-1s Inhibition Reduces the Stimulatory Potency of moDCs Toward Allogeneic T Cells

**Figure 1:**
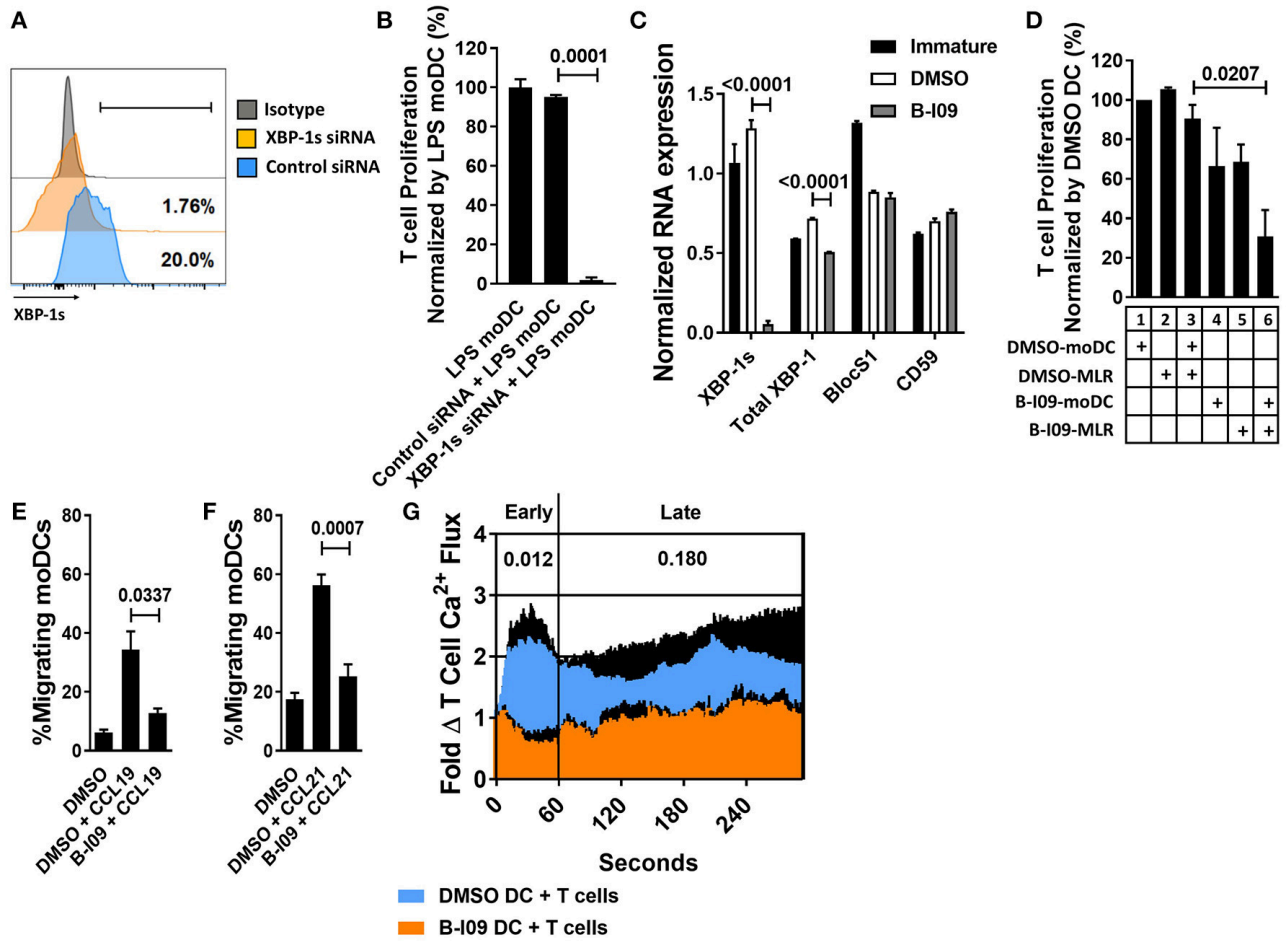
XBP-1s inhibition reduces the stimulatory potency of moDCs toward allogeneic T cells. Human moDCs were stimulated with LPS (1 μg/ml) for 24 h in the presence of XBP-1 or control siRNA. **(A)** Representative histograms show XBP-1s expression in siRNA-treated moDCs. **(B)** T cells were stimulated by XBP-1- or control-siRNA-treated moDCs in alloMLRs. T cell proliferation (MTS colormetric assay) after 5 days is shown. AlloMLRs were plated in replicates of 5 at a moDC: T cell ratio of 1:30. 1 representative experiment of 2 independent studies is shown, Dunnett's test. **(C)** Human moDCs were stimulated with LPS (1 μg/ml) for 24 h in the presence of B-I09 (20 μM) or DMSO (0.1%). Bar graph shows triplicate mean XBP-1s or total XBP-1 mRNA, vs. the RIDD components BlocS1 and CD59 in B-I09- or DMSO-treated moDCs after 24 h of LPS stimulation as measured by RT-PCR. 1 representative experiment of 4 independent studies is shown, Tukey's test. **(D)** T cell proliferation (MTS colormetric assay) measured in 5-day MLRs using B-I09- or DMSO-treated allogeneic, moDCs. Table depicts whether moDCs were pre-treated with DMSO (0.1%) or B-I09 (20 μM), or if DMSO (0.1%) or B-I09 (20 μM) was added to the MLR medium. Replicate means from 4 independent experiments are shown, Dunnett's test. **(E,F)** Human moDCs were LPS-stimulated for 24 h with B-I09 or DMSO. Bar graphs show proportion of migrating moDCs in transwell assays testing CCL19 or CCL21 (300 ng/ml, 3 h) chemotaxis. Replicate means from 4 independent experiments are shown for each, Dunnett's test. **(G)** Human moDCs were stimulated with LPS (1 μg/ml) for 24 h in the presence of B-I09 (20 μM) or DMSO (0.1%), and then used to activate allogeneic T cells for 5 days. T cells were rested for 24 h at 37°C, loaded with Fluo-4 dye for 30 min, then restimulated with fresh B-I09- or DMSO-treated moDCs during live cell imaging to monitor calcium flux in real time. Replicate means from 3 independent studies are shown. *P* values are shown for early (<60 s) and late (>60 s) T cell calcium flux after initial moDC:T cell interaction by comparing the mean AUCs after stimulation with B-I09- or DMSO-treated moDCs, paired *t-*test.

XBP-1 mRNA is constitutively spliced by IRE1α in moDCs at steady-state (14, 39). The TLR4 agonist, LPS, triggers ER stress and augments XBP-1 splicing (40). We first tested the effect of XBP-1s blockade on human moDC stimulatory capacity in alloMLRs. To genetically suppress XBP-1s, human moDCs were treated with XBP-1 or control siRNA and stimulated with LPS for 24 h (Figure 1A). Allogeneic T cells responding to the XBP-1 siRNA-treated moDCs showed a significant reduction in proliferation compared to controls (Figure 1B).

B-I09 blocks the RNAse activity of IRE1α, suppressing XBP-1 splicing (28). B-I09 (20 μM) has shown on-target XBP-1s inhibition in murine B cells and chronic lymphocytic leukemia (28). Similar to its effects on murine B cells, B-I09 significantly reduces XBP-1s in human, LPS-stimulated moDCs (Supplemental Figures 1A,B). We confirmed on-target inhibition of XBP-1s in moDCs by B-I09 via RNA expression, and verified that key regulated IRE1 dependent decay (RIDD) substrates, BlocS1 (41) and CD59 (42), were unaffected by the inhibitor compared to DMSO (Figure 1C). IRE1α also exhibits kinase activity and phosphorylates c-Jun N-terminal kinase (JNK) in response to ER stress (43). Disruption of JNK signaling could interfere with moDC function (44). However, B-I09 did not suppress IRE1α-mediated JNK phosphorylation in human moDCs (Supplemental Figures 2A,B).

Despite the inhibitory effect of B-I09 on moDC XBP-1 splicing, B-I09 did not impair LPS-mediated moDC maturation or viability (Supplemental Figures 3A–D). Pre-treating the moDCs with B-I09 (20 μM) during LPS-maturation had no significant effect on the stimulation of allogeneic T cell proliferation (Figure 1D, condition 1 vs. condition 4). Adding B-I09 to the allogeneic co-culture alone produced a modest reduction in T cell proliferation (Figure 1D, condition 2 vs. condition 5). moDC-allostimulated T cell proliferation was significantly impaired when the moDCs were first pre-treated with B-I09 during LPS-maturation and added once again to the alloMLR medium (Figure 1D, condition 3 vs. condition 6). Therefore, moDCs require XBP-1s suppression during maturation and also during their interactions with T cells to fully inhibit the alloresponse. Based on these data, allogeneic co-cultures described hereafter used either DMSO- (0.1%) or B-I09-pretreated (20 μM), LPS-stimulated moDCs. B-I09 or vehicle was also added to the MLR medium once on day 0 to maintain XBP-1s suppression during culture unless otherwise indicated.

B-I09 was not toxic toward human T cells in treated allogeneic co-cultures (Supplemental Figure 4A) and we were unable to detect significant amounts of XBP-1s in DC-allostimulated T cells (Supplemental Figure 4B). Although CD3/CD28 bead-stimulation produced significant amounts of XBP-1s in responder T cells (Supplemental Figure 4C), B-I09 had no suppressive effect on these responder T cells (Supplemental Figure 4D). Taken together, these findings indicate that the immune suppressive effect of XBP-1s inhibition acts primarily on human moDCs, impairing their allostimulatory capacity, and secondarily limits allogeneic T cells.

### XBP-1s Blockade Impairs Human moDC Migration and Induction of T Cell Calcium Flux

TLR4 facilitates the migration of moDCs by inducing CCR7 surface expression (45). In trans-well assays, B-I09 abrogates moDCs motility toward the CCR7 ligands, CCL19, and CCL21, compared to vehicle-treated cells (Figures 1E,F). T cell activation is a chief function of DCs. Despite intact potential by the allogeneic moDCs to mediate costimulation by CD86 (Supplemental Figure 3A), we identified that T cell activation via early (< 60 s) phase calcium flux was reduced in response to B-I09-treated moDCs (Figure 1G).

### XBP-1s Inhibited moDCs Reduce Th1 Differentiation

**Figure 2:**
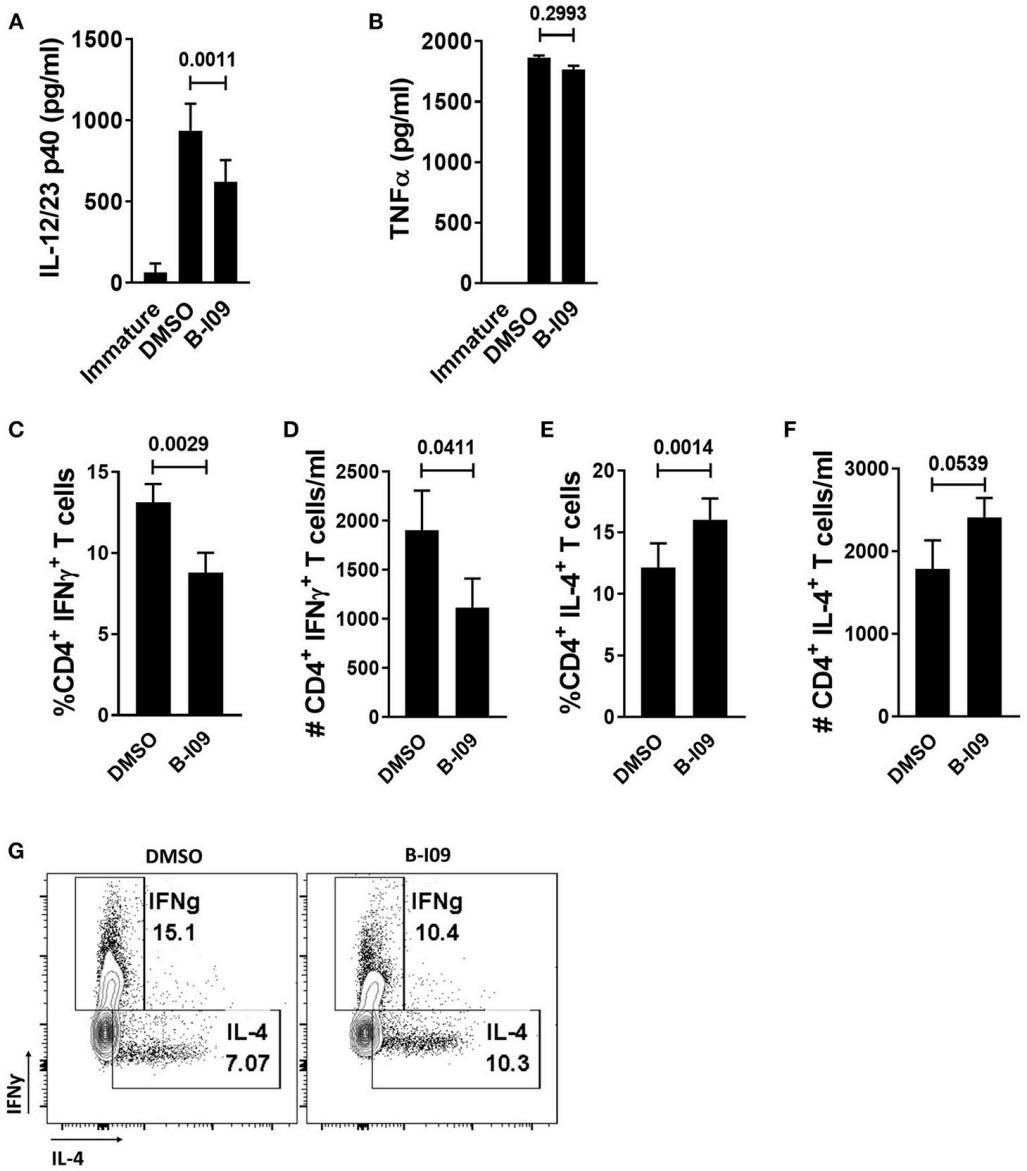
XBP-1s inhibited moDCs reduce Th1 differentiation**. (A,B)** ELISAs were used to determine the concentration of IL-12/23 p40 cytokines or TNFα in the supernatants of B-I09- or DMSO-treated moDCs after 24 h of LPS stimulation. Replicate means from 8 (IL-12/23 p40) and 2 (TNFα) independent experiments are shown, Dunnett's test. **(C–G)** T cells were cultured with B-I09 or DMSO pre-treated moDCs (moDC:T cell ratio of 1:30), and additional B-I09 (20 μM) or DMSO (0.1%) was added once on day 0. Harvested T cells were evaluated on day +5 for Th1 (CD4^+^, IFNγ^+^) and Th2 (CD4^+^, IL-4^+^) phenotype. Percentage or absolute numbers of Th1 **(C,D)** and Th2 **(E,F)** are shown. **(G)** Representative contour plots show CD4^+^ Th1 vs. Th2 cells on day +5 of the allogeneic co-culture. Means of 8 independent experiments are shown, paired t-test.

Since Th1 cells are implicated in GVHD pathogenesis (46), we investigated the effect of B-I09 on Th1 and Th2 responses *in vitro*. B-I09 reduced LPS-stimulated moDC production of p40 cytokines, implicated in Th1 differentiation (Figure 2A), without impairing moDC TNFα production (Figure 2B). T cells stimulated by allogeneic moDCs pretreated with B-I09, along with B-I09 added to the co-culture once on day 0 had a significantly reduced Th1 response compared to controls (Figures 2C,D,G). Conversely, XBP-1s blockade significantly increased the amount of Th2 cells after 5 days of culture in a proportional manner (Figures 2E-G). The shifts in Th1 were consistent with reduced p40 cytokine production by moDCs treated with B-I09.

### Targeting XBP-1s Abrogates moDC Production of IL-1β, TGFβ, and Diminishes Alloresponder Th17 Differentiation

**Figure 3:**
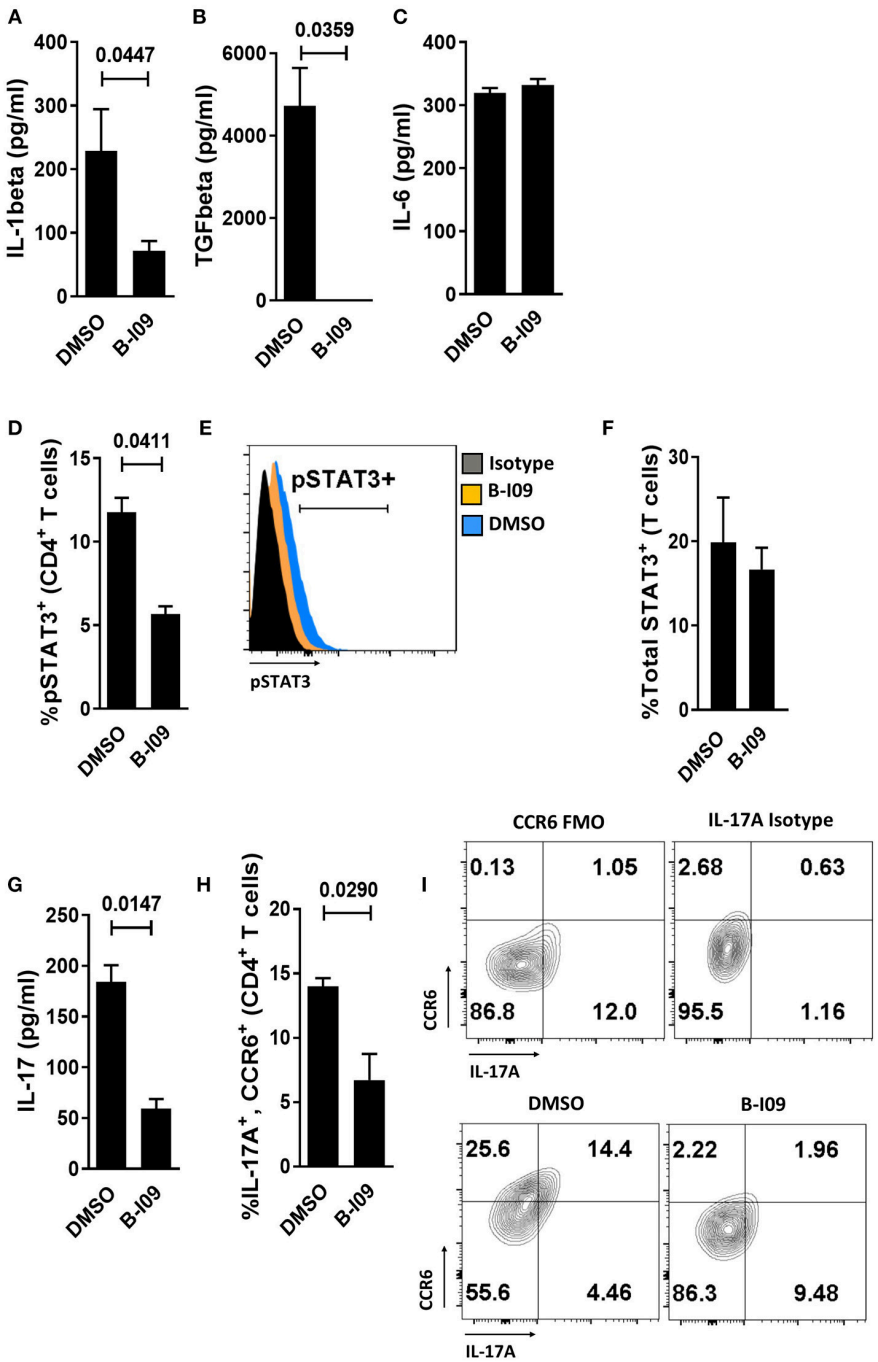
Targeting XBP-1s abrogates human moDC production of IL-1β, TGFβ, and diminishes alloresponder Th17 differentiation**. (A–C)** Supernatant concentrations of IL-1β, TGFβ, or IL-6 from LPS-stimulated moDCs exposed to B-I09 or DMSO after 24 h of culture are shown. Replicate means from 5 (IL-1 β), 3 (TGFβ), and 4 (IL-6) independent experiments are shown, paired *t*-test. **(D–H)** T cells were cultured with B-I09 or DMSO pre-treated moDCs (moDC:T cell ratio of 1:30), and additional B-I09 (20 μM) or DMSO (0.1%) was added once on day 0. pSTAT3^+^ CD4^+^ T cells were analyzed at day +5 by flow cytometry **(D)** and representative histograms are shown **(E)**. Means from 3 independent experiments are shown, paired *t*-test. **(F)** Total STAT3 was measured in T cells from co-cultures of DCs and T cells treated with B-I09 or DMSO. Means from 3 experiments are shown. In similarly treated co-cultures, the supernatant concentration of IL-17 was quantified **(G)** and Th17 (CD4^+^, CCR6^+^, IL-17A^+^) differentiation was evaluated by flow cytometry **(H,I)**. Replicate means from 3 (IL-17 ELISA) and 4 (Th17) independent experiments, paired *t*-test. **(I)** Representative contour plots are shown.

We next investigated whether B-I09 could reduce Th17-inducing cytokines by LPS-stimulated moDCs. XBP-1s blockade significantly suppressed IL-1β production by human moDCs (Figure 3A) and eliminated TGFβ from the supernatant of LPS-stimulated moDCs (Figure 3B). IL-1β is an essential co-factor for IL-6 signal transduction (47). Despite ample IL-6 production by B-I09- or DMSO-treated moDCs (Figure 3C), CD4^+^ T cells co-cultured with these moDCs in the presence of B-I09 displayed significantly less STAT3 phosphorylation (Figures 3D,E). Total STAT3 expression within T cells stimulated by B-I09- or DMSO-treated moDCs was similar (Figure 3F). Moreover, Th17 differentiation was significantly decreased by XBP-1s inhibition (Figures 3G–I) and partially rescued by adding IL-1β, but not TGFβ, to B-I09 treated co-cultures (Supplemental Figure 5).

### moDC XBP-1s Directs iTreg Differentiation via TGFβ

**Figure 4:**
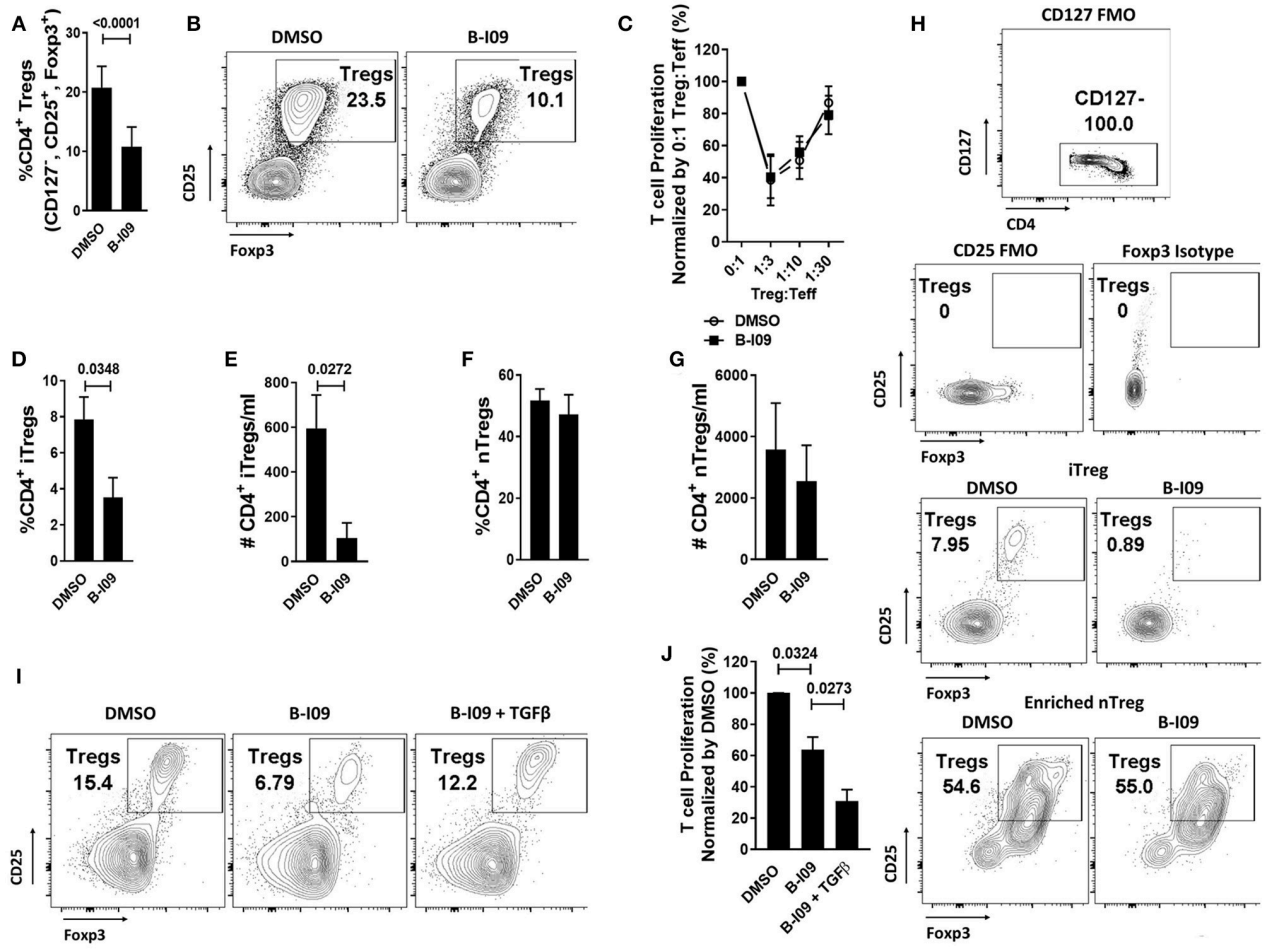
moDC XBP-1s direct human iTreg differentiation via TGFβ. T cells were cultured with B-I09 or DMSO pre-treated moDCs (moDC:T cell ratio of 1:30), and additional B-I09 (20μM) or DMSO (0.1%) was added once on day 0. **(A)** Percentage of Tregs (CD4^+^, CD127^−^, CD25^+^, Foxp3^+^) in the 5-day co-cultures, with representative contour plots shown **(B)**. Means from 6 independent experiments are shown. **(C)** The suppressive capacity of harvested moDC-allostimulated Tregs was tested at different ratios of Treg to T cell responders stimulated by fresh allogeneic moDCs (moDC:responder T cell ratio 1:30) in alloMLRs. No additional B-I09 or DMSO was added. Graph shows mean percent T effector (Teff) proliferation measured by Ki-67. Means from 3 independent experiments are shown. To generate inducible Tregs, Treg-depleted CD4^+^ Tconv were stimulated by B-I09 or DMSO-pretreated allogeneic moDCs at a moDC:T cell ratio of 1:30. B-I09 or DMSO was added once on day 0. Magnetic bead enriched natural Tregs (CD4^+^, CD25^+^) were similarly treated in allogeneic Treg:moDC co-cultures. Treg populations were evaluated by flow cytometry on day +5. Means from 4 iTreg and 4 nTreg independent experiments are shown, paired *t*-test. Percentage and absolute number of iTreg **(D,E)** and nTreg **(F,G)** are shown. **(H)** Representative contour plots are shown for iTreg and nTreg. **(I)** Representative contour plots show that adding recombinant human TGFβ rescues iTreg generation in Treg-depleted alloMLRs treated with B-I09 vs. DMSO. 1 representative experiment of 2 independent studies is shown. **(J)** T cell proliferation (MTS colormetric assay) at day +5 among Treg-depleted alloMLRs treated with B-I09 or DMSO, with recombinant human TGFβ added as indicated. Replicate means from 4 independent experiments are shown, Dunnett's test.

As XBP-1s-inhibited moDCs impaired Th17 differentiation, we were surprised to observe that such moDCs also significantly reduced responder Treg frequency in treated alloMLRs (Figures 4A,B), though the suppressive function of antigen-specific Tregs remained intact (Figure 4C). We then investigated whether the reduction in Tregs stimulated by B-I09-treated moDCs was due to impaired differentiation of iTregs or suppression of nTregs. Treg-depleted Tconv or purified nTregs were cultured with DMSO- or B-I09-pretreated, allogeneic moDCs, and DMSO or B-I09 was also added to the media, respectively. XBP-1s inhibition significantly reduced both the frequency and absolute number of iTregs stimulated by allogeneic moDCs (Figures 4D,E,H) in contrast to nTregs (Figures 4F,G,H). Based on these data, we surveyed known moDC-mediated mechanisms for iTreg generation. The expression of indolamine 2,3-deoxygenase (48) was similar among B-I09- or DMSO-treated moDCs (Supplemental Figures 6A,B), as was STAT5 phosphorylation among the DC-allostimulated T cells (Supplemental Figures 6C,D). Instead, we discovered that exogenous TGFβ rescued the differentiation of iTregs in the co-cultures containing XBP-1s-inhibited moDCs (Figure 4I). Moreover, adding TGFβ to Treg-depleted, MLRs stimulated by B-I09-treated moDCs provided even greater suppression of allogeneic T cells (Figure 4J). These data suggest that XBP-1s-mediated TGFβ production by human moDCs contributes to allogeneic iTreg differentiation, and that moDC XBP-1s activity is not required for Treg suppressive potency or nTreg responses.

### XBP-1s Is Dispensable for Anti-tumor Activity by CD8^+^ Cytotoxic T Lymphocytes and NK Cells

**Figure 5:**
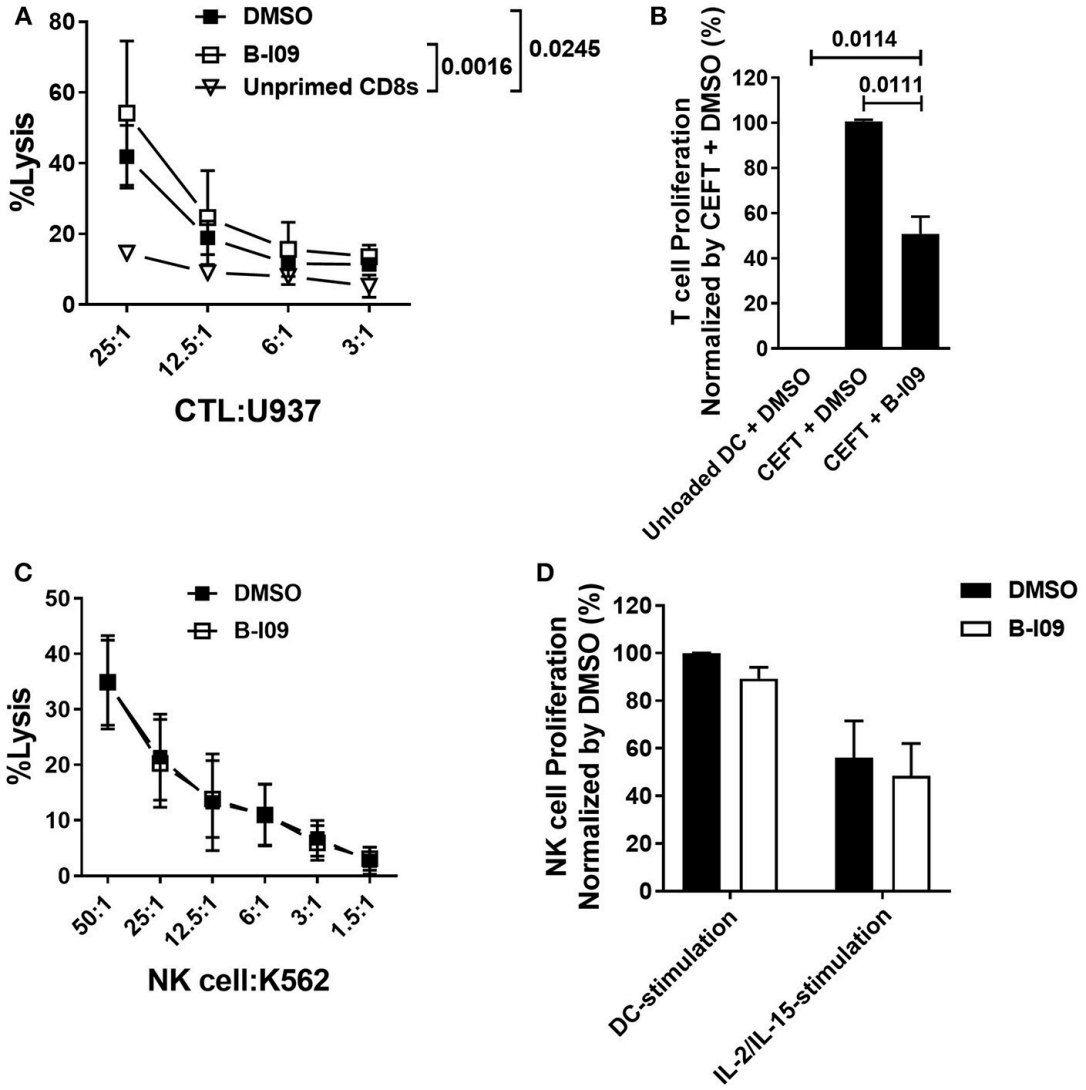
XBP-1s is dispensable for anti-tumor activity by CD8^+^ cytotoxic T lymphocytes and NK cells. **(A)** Replicate mean specific lysis by human CD8^+^ CTL generated *in vitro* using PBMCs stimulated by irradiated U937cells (1:1) on days 0 and +7 of a 10–12 day culture. B-I09 or DMSO was added once on days 0 and +7. Tumor-specific killing by purified CD8^+^ T cells was determined using fresh U937 cells. U937 lysis was measured by a colorimetric assay after 4 h. Triplicate means from 3 independent experiments are shown. **(B)** T cell proliferation stimulated by autologous moDCs loaded with CMV, EBV, influenza, or tetanus peptides is shown. B-I09 or DMSO was added once on day 0. T cell proliferation (MTS colormetric assay) was measured on day +3. Replicate means from 4 independent experiments are shown, Dunnett's test. **(C)** Mean specific lysis by human NK cells against K562 targets is shown. B-I09 or DMSO was added at the outset of the culture. K562 lysis was measured by a colorimetric assay after 4 h. Replicate means from 3 independent experiments are shown. **(D)** NK cell proliferation stimulated by allogeneic moDCs (moDC:NK cell ratio 1:10) or IL-2 plus IL-15. NK cell proliferation (MTS colormetric assay) was measured on day +5. Replicate means from 3 independent experiments are shown.

While inhibiting moDC-XBP-1s in alloMLRs impaired the proliferation of responder T cells, CTLs generated with B-I09-treated stimulators exhibited intact tumor specific lytic function (Figure 5A). Compared to unloaded moDCs, T cell responses to clinically relevant pathogens using peptide-loaded, B-I09 exposed autologous moDCs permitted a measureable response to CMV, EBV, influenza, and tetanus albeit significantly less robust than DMSO-treatment of peptide-loaded DCs (Figure 5B). In evaluating the effect of XBP-1s-inhibited moDCs on responder T cell memory phenotypes after 5 days of stimulation, we identified that blocking the moDC ER stress response increased the proportion of non-alloreactive, naïve CD8^+^ T cells vs. alloreactive central and effector memory CD8^+^ T cells compared to vehicle controls (Supplemental Figures 7A,B). The proportion of responder CD4^+^ T cell memory subsets was similar regardless of B-I09- or DMSO-treated moDC-stimulation (Supplemental Figures 7C,D).

NK cells and moDCs both constitutively express XBP-1s (49). The functional significance of XBP-1s in NK cells is not known. Because NK cells can mediate important anti-tumor effects after allo-HCT, we investigated the effect of B-I09 on NK cell lytic capacity and proliferative responses. Human NK cells readily destroyed K562 targets in the presence of B-I09 or DMSO (Figure 5C). Despite their exposure to B-I09 NK, cells proliferated when stimulated by allogeneic, immature moDCs or a cocktail of IL-2 and IL-15 (Figure 5D).

### XBP-1s Blockade Reduces Human Skin Graft Rejection and Xenogeneic GVHD, Yet Preserves *in vivo* Generation of Anti-tumor CTL

**Figure 6:**
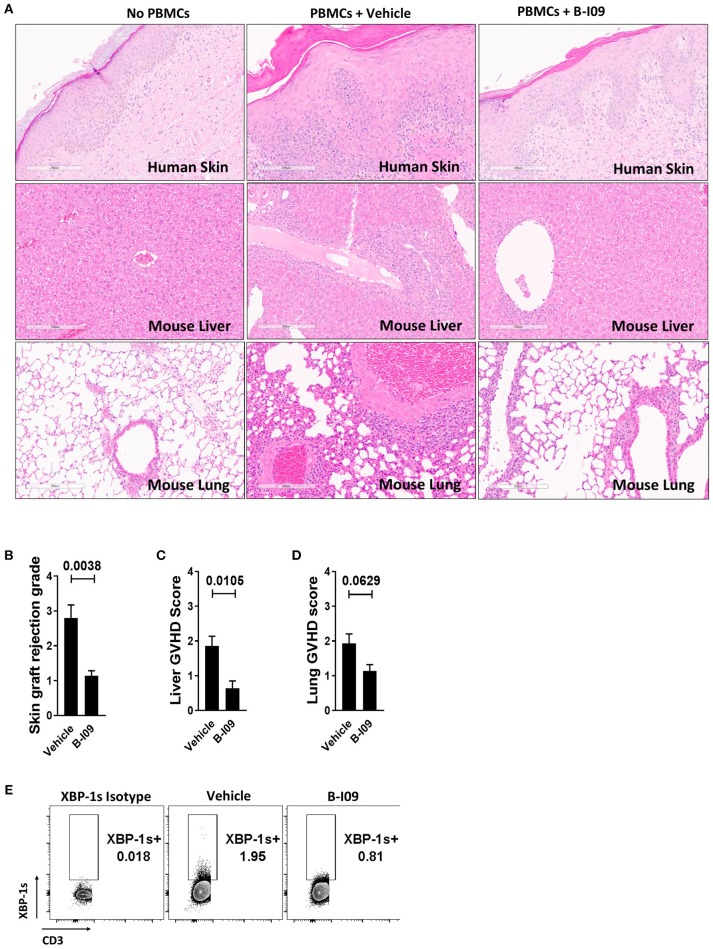
XBP-1s blockade reduces human skin graft rejection and xenogeneic GVHD. NSG mice received a 1 cm^2^ split thickness human skin graft. After 30 days of rest to permit engraftment, 5 × 10^6^ human PBMCs (allogeneic to the skin) were injected into the mice. Unique pairs of donor skin and allogeneic PBMCs were used for each set of experiments. B-I09 30 mg/kg or vehicle was given by i.p. injection 5 days a week for 3 weeks. Mice were humanely euthanized and the skin graft, spleen, lung, and liver were harvested from the recipient on day +21 from time of PBMC injection. **(A)** Representative H&E sections compare skin graft rejection and xenogeneic GVHD in the liver and lung among no PBMC controls, mice that received PBMCs plus vehicle, and mice that received PBMCs plus B-I09 (100X). **(B–D)** Bar graphs show skin graft rejection and xenogeneic GVHD scores (blinded assessment) at day +21. **(E)** Representative contour plots show the amount of detectable XBP-1s in CD3 negative cells residing in the murine spleen at day +21. Pooled data from two independent experiments, up to 7 mice per group, Mann–Whitney test.

**Figure 7:**
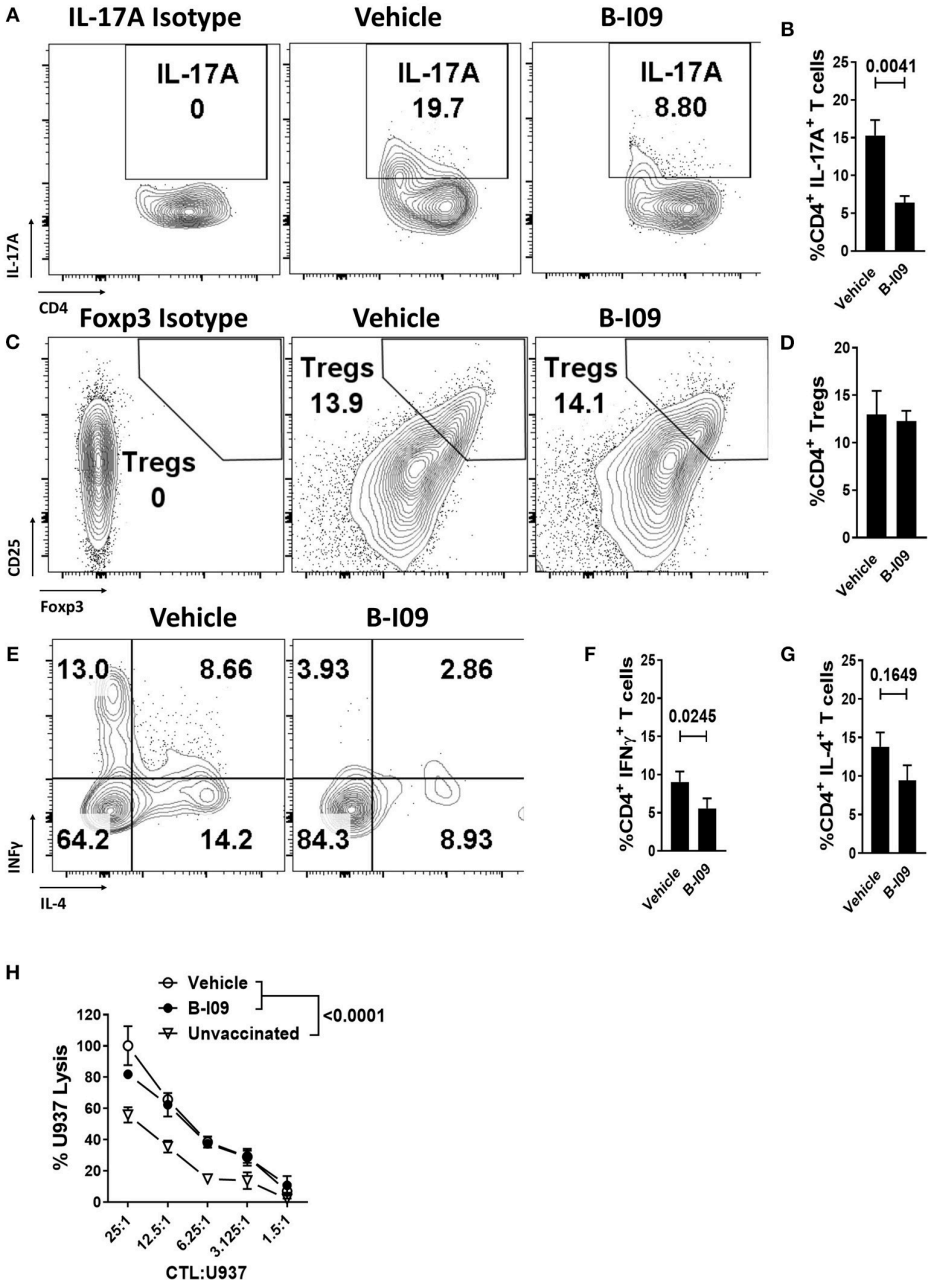
XBP-1s inhibition reduces pathogenic Th17 and Th1 cells, yet preserves generation of anti-tumor CTL and Tregs *in vivo*. NSG mice were transplanted with human skin grafts and allogeneic PBMCs and treated with vehicle or B-I09 exactly as described. On day +21, the mouse spleens were harvested and human T cell phenotypes were determined by flow cytometry. The amount of human **(A,B)** Th17s (CD4^+^, IL-17A^+^), **(C,D)** Tregs (CD4^+^, CD127^−^, CD25^+^, Foxp3^+^), Th1s (CD4^+^ IFNγ^+^), and Th2s (CD4^+^, IL-4^+^) **(E–G)** isolated from the recipient spleen at day +21 are shown. Pooled data from two independent experiments, up to 7 mice per group, Mann-Whitney test. **(H)** Replicate mean specific lysis by human CD8^+^ CTL generated *in vivo* using NSG mice transplanted with human PBMCs (30 × 10^6^) and vaccinated with irradiated U937cells (10 × 10^6^) on days 0 and +7. Mice received B-I09 or vehicle as described. For these experiments, recipients did not receive human skin. U937 lysis was measured by a colorimetric assay after 4 h using purified human CD8^+^ T cells from recipient spleens at days +10–12. Replicate mean tumor lysis values shown are from 1 of 2 independent experiments, Tukey's test.

Skin is a highly immunogenic tissue and a critical target-organ in GVHD (1). To test the efficacy of B-I09 *in vivo* using human immune cells, NSG mice were transplanted with a human skin xenograft using a well-established model (33, 36). After 30 days to heal, mice were later injected with 5 × 10^6^ human donor PBMCs allogeneic to the skin to induce graft rejection and xenogeneic GVHD (33, 36). B-I09 or vehicle was administered at 30mg/kg by intraperitoneal injection 5 days a week for 3 weeks. XBP-1s blockade significantly reduced skin graft rejection by the allogeneic PBMCs, as measured on day +21 after the adoptive transfer of allogeneic PBMCs (Figures 6A,B). Importantly, B-I09 also significantly reduced xenogeneic GVHD by the human T cells against murine liver (Figures 6A,C) and modestly against mouse lungs that did not quite reach statistical significance (Figures 6A,D). *In vivo* XBP-1s inhibition by B-I09 could be seen in CD3 negative cells isolated from the recipient spleens (Figure 6E). Consistent with *in vitro* observations, B-I09 significantly reduced the amount of human Th17 cells (CD4^+^, IL-17A^+^) in the mouse spleens (Figures 7A,B). Human Tregs (CD4^+^, CD127^−^, CD25^+^, Foxp3^+^) recovered from the host spleens were similar among B-I09- or vehicle-treated mice (Figures 7C,D). While B-I09-treated mice demonstrated a significant decrease in Th1 cells (CD4^+^, IFNγ^+^), Th2 cells (CD4^+^, IL-4^+^) were not increased as observed *in vitro* (Figures 7E-G).

We used an established method to generate human anti-tumor CTL *in vivo* and then test their specific killing (26, 33, 50). NSG mice received human PBMCs (30 × 10^6^) and were inoculated with irradiated U937 cells (10 × 10^6^) on days 0 and +7. Mice were treated with B-I09 or vehicle as described above, and human CD8^+^ T cells were isolated from the spleens of euthanized recipients during days +10-12. CD8^+^ CTLs from B-I09-treated mice demonstrated tumor killing equal to CTLs from vehicle-treated recipients, and both were significantly more potent than CD8^+^ CTLs from unvaccinated controls (Figure 7H). Preliminary data from a pilot cohort of patients undergoing allogeneic HCT (5 with acute GVHD and 5 without), suggests that the amount of intracellular XBP-1s is significantly increased in CD1b^+^, epidermal DCs among skin biopsies from patients with acute GVHD compared to no GVHD controls (Supplemental Figures 8A–C). Though not statistically significant, we also observed a trend toward increased CD1b^+^, XBP-1s^+^epidermal DCs in the GVHD cohort (Supplemental Figure 8B). This observation among a limited number of patients is provocative and merits the prospective study of XBP-1s^+^ DCs in acute GVHD pathogenesis.

## Discussion

We demonstrated that blocking XBP-1s in human moDCs significantly reduced IL-1β and IL12/23p40 production during their interaction with donor T cells, suppressing Th1 and Th17 differentiation. While XBP-1s inhibition restrained T cell alloreactivity, anti-tumor responses by CTLs and NK cells remained intact. While rodent models have shown targeting XBP-1s in B cells reduces chronic GVHD without impairing graft- vs.-leukemia (GVL) effects (25), we now demonstrate that suppressing XBP-1s in human DCs limits acute GVHD without impairing donor immunity toward cancer. We also detected a partial response toward clinically relevant infectious antigens by T cells stimulated by XBP-1s-inhibited, peptide loaded moDCs.

We showed XBP-1s inhibition protected human skin grafts from alloreactive T cell rejection *in vivo*. Xenogeneic GVHD mediated by human T cells was also decreased by targeting XBP-1s. Altogether, our data show that XBP-1s is a relevant therapeutic target to suppress key aspects of DC function and prevent GVHD after alloHCT.

Our results are consistent with prior studies which demonstrate that eliminating ASC or the NLRP3 inflammasome in bone marrow transplant recipients significantly reduces GVHD in mice (15). Our approach substantially extends these data by interrogating XBP-1s in human cells and demonstrating regulation of NLRP3 activation in the context of ER stress (9, 51). While targeting either the inflammasome or XBP-1s reduces Th17 differentiation (15), XBP-1s inhibition differs from NLRP3 blockade by also significantly reducing Th1 and iTreg responses. This defect in moDC supported human iTreg generation is correctable by adding exogenous TGFβ, which is otherwise absent from B-I09-treated moDCs. We show XBP-1s blockade does not diminish the number or frequency of allo-stimulated nTregs or total Treg suppressive potency. Interestingly, XBP-1s inhibition was not detrimental to human Tregs *in vivo*. In the xenogeneic transplant experiments, recipient mice were transplanted with whole human PBMCs. Though alloMLRs are restricted to T cells and moDCs, we speculate the more diverse non-moDC constituents of the PBMC inoculum may potentially rescue Tregs *in vivo* by providing TGFβ (52). Additionally, the human and murine TGFβ1 genes share 66% nucleotide homology and it is possible that cross-reactive murine TGFβ could also rescue the human iTregs *in vivo* (53).

Targeting XBP-1s with B-I09 or siRNA supports translation of our proposed strategy in GVHD prevention. While we show that anti-tumor responses by CTLs and NK cells remain intact *in vitro*, we acknowledge that these experiments do not fully replicate the biology of GVL *in vivo*. Despite this limitation, our study design demonstrates the relevance of DC XBP-1s in human GVHD and that targeting XBP-1s does not impair donor anti-tumor immunity. We surmise that the ability of the DC to express critical T cell costimulatory molecules, such as CD86, despite XBP-1s inhibition is important for the preserved GVL effect. The essential role for CD86 in CD8 CTL-mediated tumor clearance is well-demonstrated (54–56). Furthermore, the blunting of GVHD is likely driven by impaired DC production of IL-1β and reduced differentiation of pathogenic Th1 and Th17 cells by XBP-1s blockade.

Silencing XBP-1 in intratumoral suppressive DCs enhances T cell responses to cancer antigens, a result that is distinct from our observations in GVHD (57). It is reasonable that ER stress mediators support antigen-presentation by DCs during acute GVHD. In contrast, sustained, unremitting ER stress in tumor bed DCs, along with associated metabolic alterations (57), abrogates their immunostimulatory activity. For example, in ovarian cancer, ER stress leads to lipid peroxidation, impaired antigen presentation, and blunted stimulatory capacity toward responder T cells (57). Alternatively, context-dependent ER stress effects could depend on tumor or GVHD target-organ location, and/or be influenced by a different cytokine milieu altogether. Understanding the context-dependent effects of ER stress in cancer vs. inflammation is an area of active interest.

The ER stress response of DCs represents a novel biologic target to prevent GVHD in humans. In summary, targeting DC XBP-1s is an innovative approach to selectively impair alloreactive T cells and pathogenic Th1/Th17 differentiation, while maintaining donor immune mediated anti-leukemia responses, that deserves consideration for clinical trials of acute GVHD prophylaxis. Additionally, a prospective investigation of epidermal XBP-1s^+^, CD1b^+^ DCs and their potential involvement in the pathogenesis of acute GVHD warrants future study.

## Author Contributions

BB designed and performed experiments, analyzed and interpreted data, and wrote the manuscript. KW, MM, JR, and AS performed experiments and edited the manuscript. FL, JP, JD, and BRB. discussed experimental design, analyzed and interpreted data, and edited the manuscript. ES scored skin graft rejection, analyzed, and interpreted tissue data, and edited the manuscript. ML and JVK harvested skin grafts and edited the manuscript. AL, JF, JK, CL, PR, and JC-G discussed experimental design and edited the manuscript. JRD, CK, C-HT, and C-CH discussed experimental design, performed experiments, synthesized B-I09, interpreted chemical analysis, and edited the manuscript. CA designed experiments, interpreted data, and edited the manuscript.

### Conflict of Interest Statement

The authors declare that the research was conducted in the absence of any commercial or financial relationships that could be construed as a potential conflict of interest.

## Abbreviations

GVHD: graft-vs.-host disease
HCT: hematopoietic cell transplantation
moDC: monocyte-derived dendritic cell
ER: endoplasmic reticulum
CTL, stress: cytotoxic T lymphocytes
LPS: lipopolysaccharide
GVL: graft- vs.-leukemia.
