# Supplementary material for: Inhibition of Human Dendritic Cell ER Stress Response Reduces T Cell Alloreactivity Yet Spares Donor Anti-tumor Immunity
Source: Front Immunol. 2018 Dec 6;9:2887. doi: 10.3389/fimmu.2018.02887 (PMC6291501; doi:10.3389/fimmu.2018.02887)
Supplement: Supplementary file 1 [file Data_Sheet_1.pdf]

Supplemental Table 1: Antibodies and Kits used

Name:	Fluor:	Manufacturer:	Catalog #:
CD3	FITC	ebioscience	11-0037-42
CD4	FITC	ebioscience	11-0048-42
CD4	PeCy7	ebioscience	25-0047-42
CD8	PE	BDbioscience	555635
CD25	PeCy7	BDbioscience	557741
CD45RO	FITC	BDbioscience	555492
CD83	PE	BDbioscience	561959
CD86	APC	BDbioscience	560956
HLA-DR	APC	ebioscience	17-9956-41
CD127	AF647	BDbioscience	558598
CCR6(CD196)	PerCP-Cy5.5	BDbioscience	560487
CD62L	PeCy7	Biolegend	304822
Ki67	PE	ebioscience	12-5699-42
Foxp3	PE	BDbioscience	560046
IFN-y	FITC	BDbioscience	554700
IL-4	APC	ebioscience	17-7049-42
IL-17A	BV421	Biolegend	11-7179-81
XBP1-S	AF647	BDbioscience	562821
pSTAT3 (Y705)	PE	BDbioscience	612569
pSTAT5 (Y694)	AF647	BDbioscience	612599
IDO	AF647	Biolegend	654003
LDH Cytotox Assay		Pierce/Thermofisher	88953
IL-1beta ELISA		Thermofisher	EH2IL1B
IL-17 ELISA		Thermofisher	88-7176-22
TGFbeta ELISA		LifeTech	BMS2494

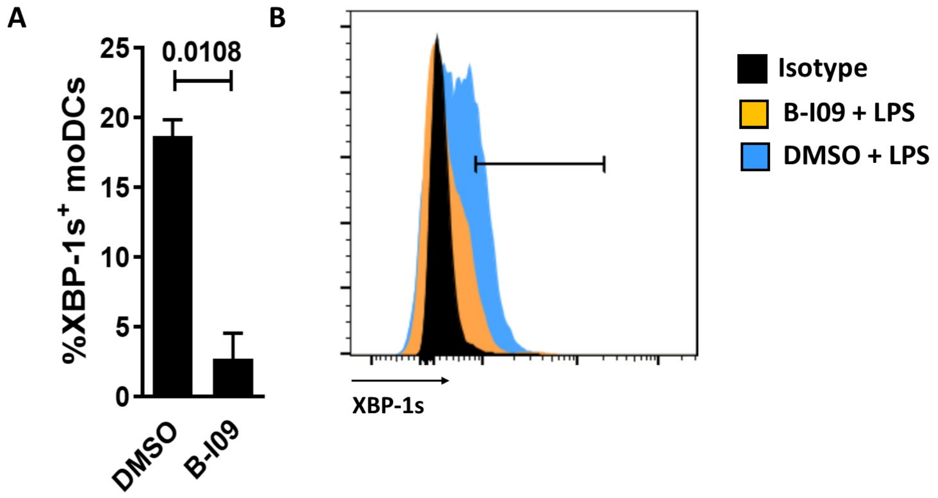

Supplemental Figure 1: B-I09 reduces XBP-1s expression in human moDCs. Human moDCs were stimulated with LPS (1μg/ml) for 24 hours in the presence of B-I09 (20μM) or DMSO (0.1%). A, B) Amount of XBP-1s^{+} moDCs after LPS stimulation among each treatment group. Representative contour plots are shown. n=3 independent experiments, paired t-test.

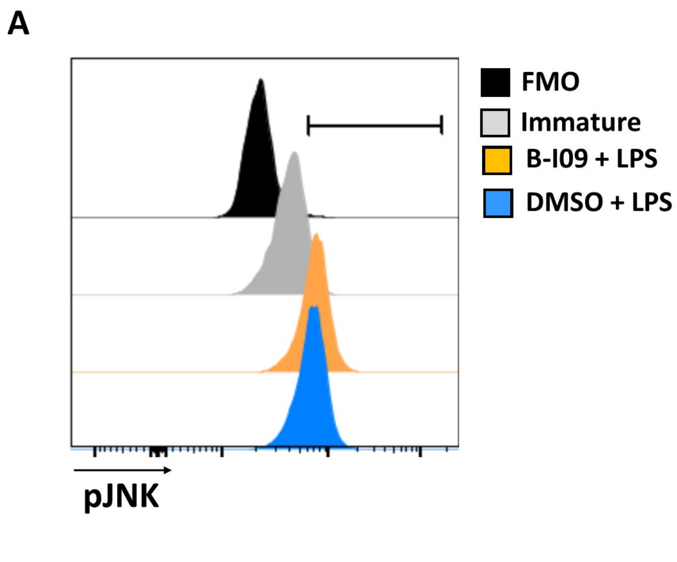

Supplemental Figure 2: B-I09 does not inhibit LPS-mediated JNK phosphorylation in human moDCs. Human monocyte-derived DCs were stimulated with LPS (1µg/ml) for 25 minutes. DMSO vehicle control (0.1%) or B-I09 (XBP1 inhibitor, 20µM) was added to the serum-free culture medium. moDCs were then harvested, fixed and permeabilized, then stained for CD83 and phospho-JNK expression. A) Representative contour plots are shown. B) graph shows the amount of $pJNK^+$ moDCs after LPS stimulation. n=3 independent experiments, Dunnett's test.

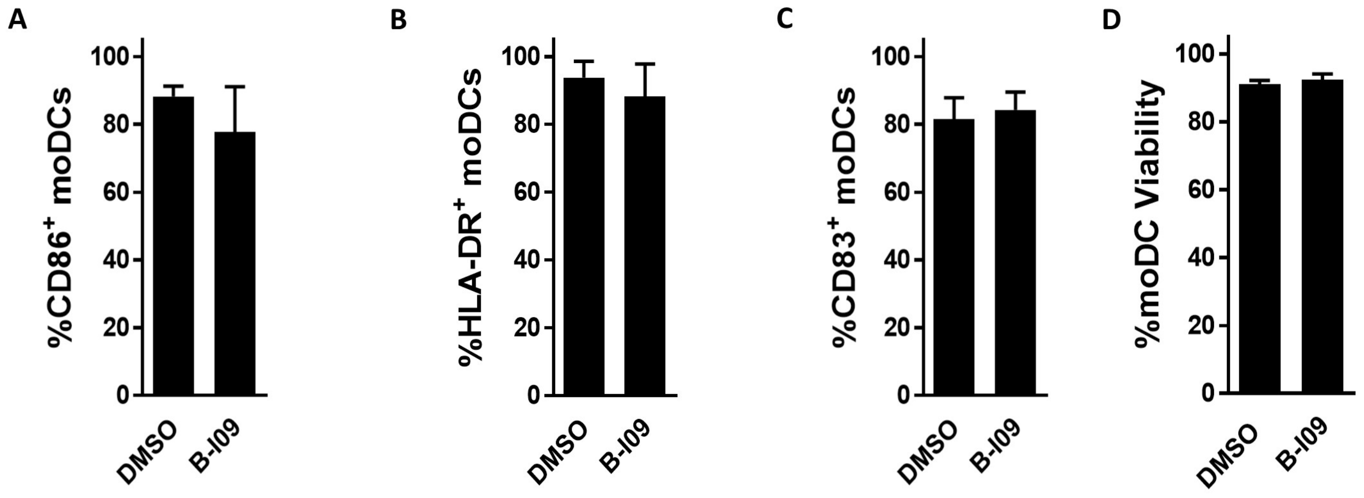

Supplemental Figure 3: Effect of B-I09 on LPS-induced moDC maturation phenotype and viability. A-D) moDC expression of CD86, HLA-DR, CD83, and moDC viability (Live/Dead Yellow) after LPS stimulation (24 hours) while treated with DMSO (0.1%) of B-I09 (20µM) are shown. n=3 independent experiments.

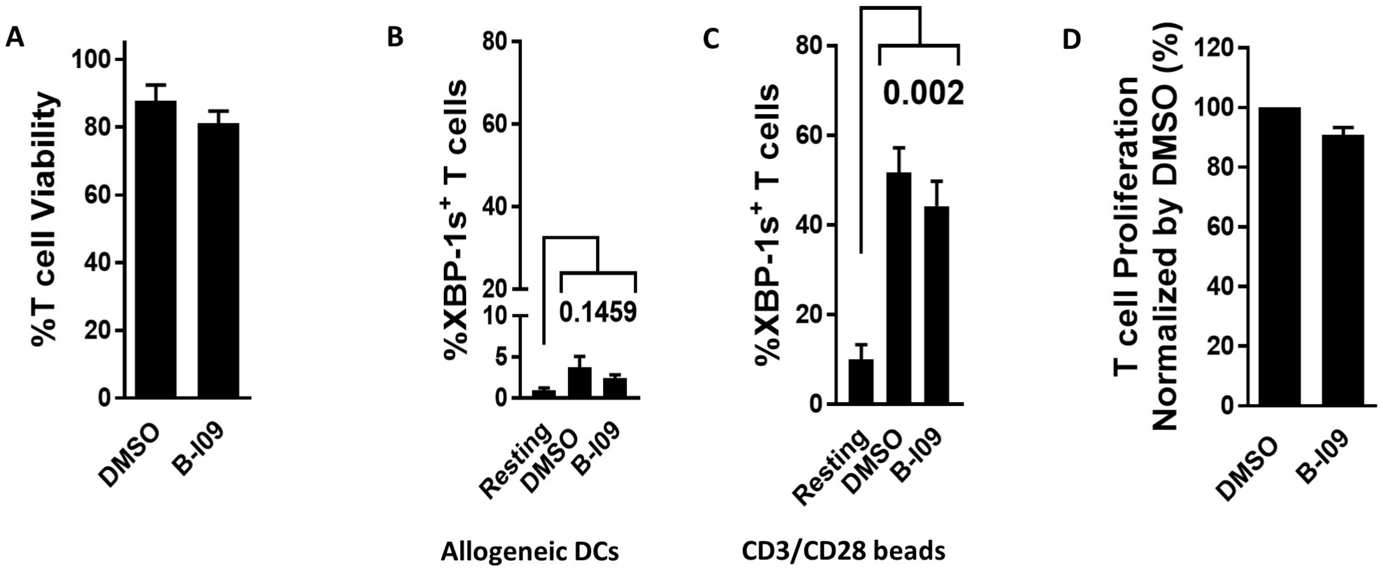

Supplemental Figure 4: Effect of B-I09 on T cell viability, XBP-1s expression, and polyclonal proliferative response. A) T cell viability measured by flow cytometry (Live/Dead Yellow) from 5-day alloMLRs including DMSO pre-treated moDCs with DMSO added to the MLR medium or B-I09 pre-treated moDCs with B-I09 added to the MLR medium. n=8 independent experiments. B) T cell XBP-1s expression after a 5 day stimulation by allogeneic moDCs pre-treated with B-I09 or DMSO during LPS-maturation with B-I09 or DMSO added to the culture medium on day 0, or C) 3 days of CD3/CD28 beads with B-I09 or DMSO added to the medium on day 0. n=3 independent experiments for each, Dunnett's test. D) T cell proliferation after 3 days of stimulation with CD3/CD28 beads while exposed to B-I09 or DMSO added once on day 0. n=3 independent experiments performed in triplicate.

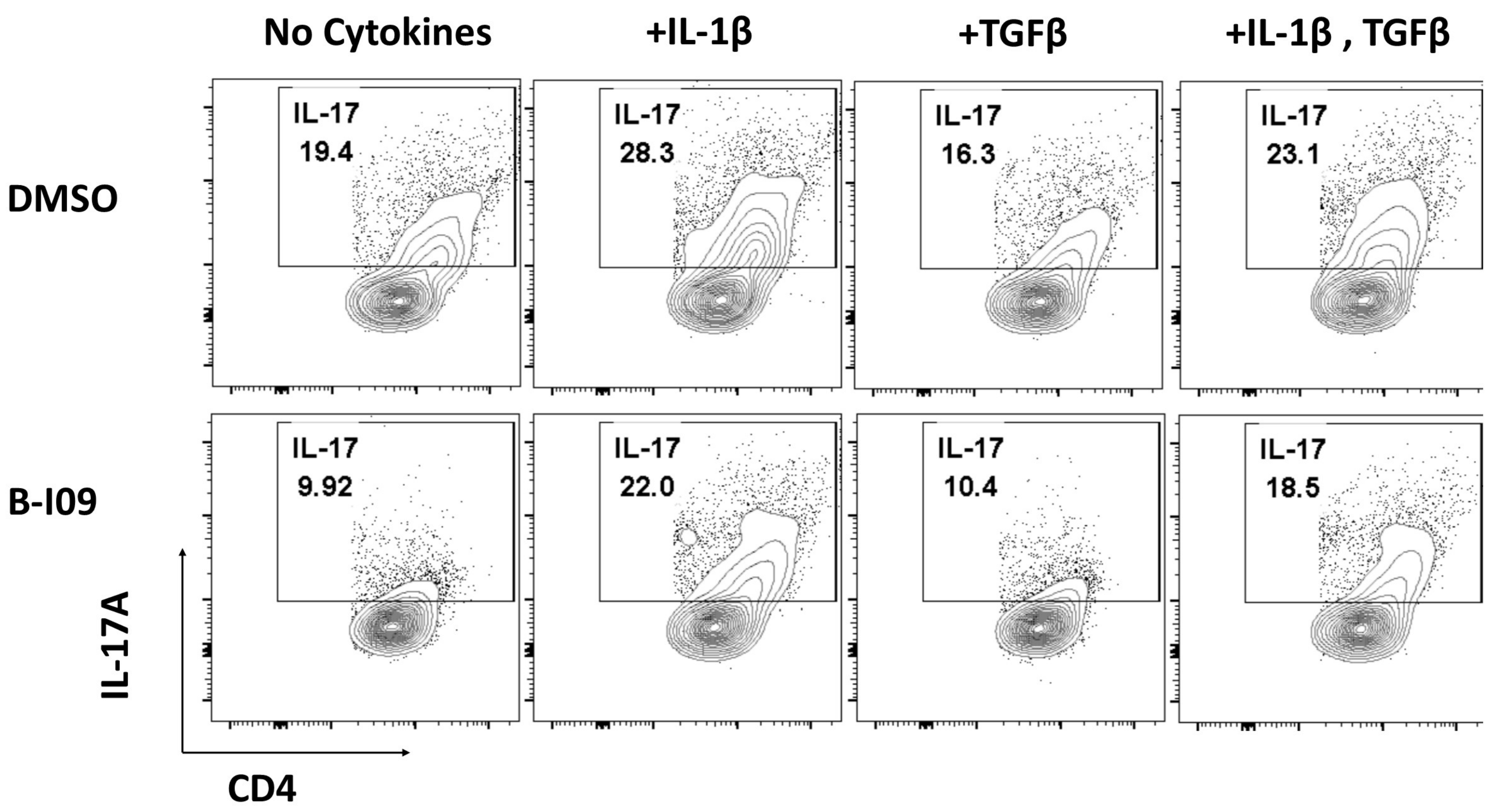

Supplemental Figure 5: Exogenous IL-1β rescues Th17 differentiation after stimulation with moDCs pretreated with B-I09. Exogenous IL-1β (10ng/ml added on days 0, +2, and +4), but not TGFβ (4ng/ml), rescues Th17 differentiation among T cells cultured with B-I09- or DMSO -pre-treated moDCs. B-I09 or DMSO was also added once on day 0 to the alloMLR. Contour plots at day +5 from 1 of 2 representative experiments is shown.

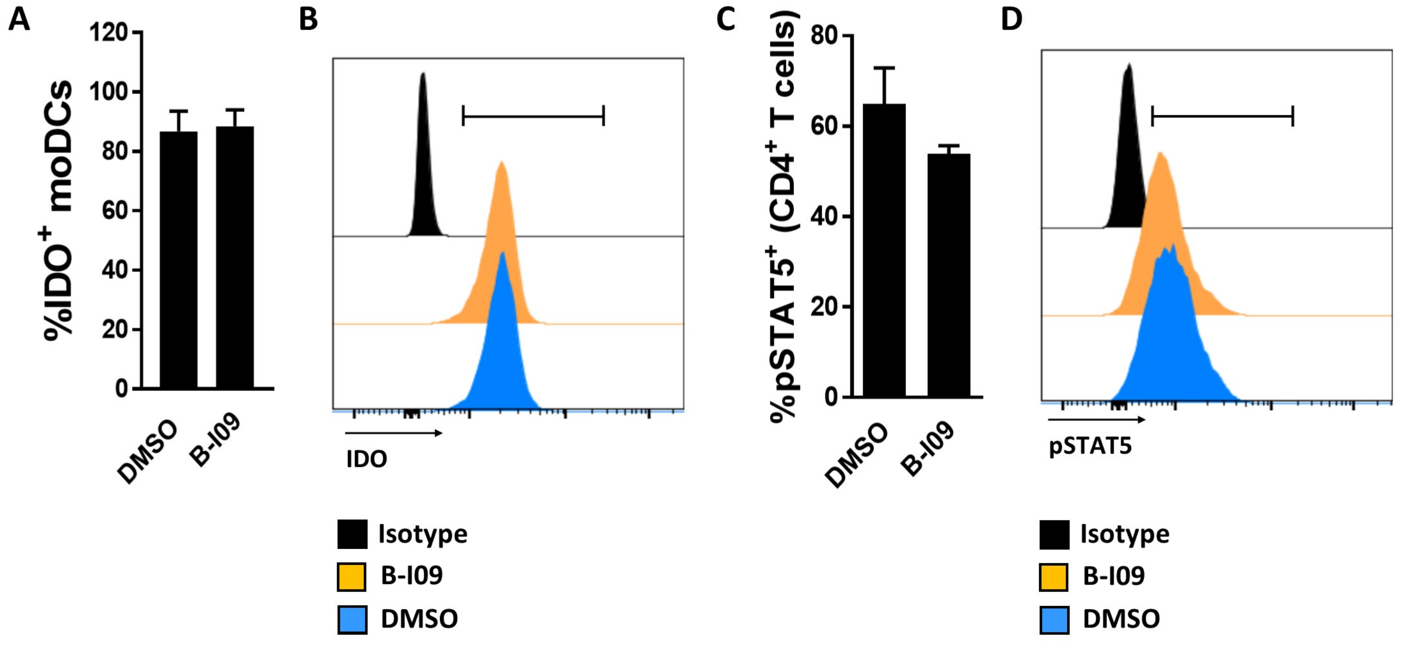

Supplemental Figure 6: Effects of XBP-1s inhibition on moDC indolamine 2,3-deoxygenase and T cell STAT5 phosphorylation. Human monocyte-derived DCs were stimulated with LPS for 24 hours in the presence of B-I09 (XBP-1s inhibitor, 20μM) or DMSO control (0.1%). moDCs were then harvested, fixed, permeabilized, and stained for intracellular indolamine 2,3-deoxygenase (IDO). Expression was analyzed by flow cytometry. A) Bar graph shows mean ± SEM IDO expression. B) representative histogram shows moDC IDO expression (gray = isotype, black line = DMSO, red line = B-I09). n=4 independent experiments. C,D) T cells were cultured with B-I09 or DMSO pre-treated moDCs (DC:T cell ratio of 1:30), and additional B-I09 (20μM) or DMSO (0.1%) was added once on day 0. $pSTAT5^{+}$ $CD4^{+}$ T cells (mean ± SEM) were analyzed at day +5 by flow cytometry as depicted in the (C) bar graph and (D) representative histograms. n=3 independent experiments.

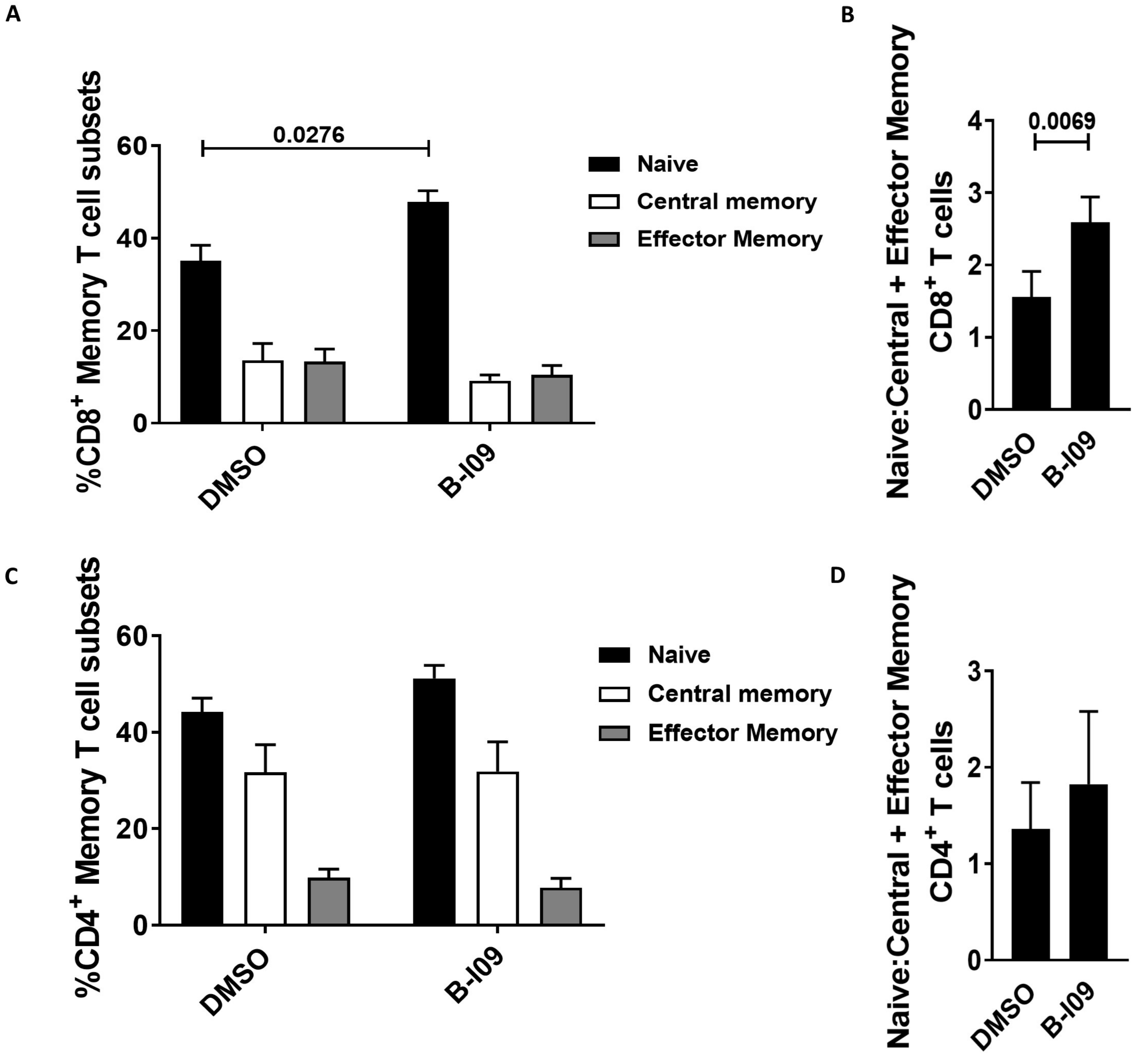

Supplemental Figure 7: Inhibiting moDC ER stress increases the proportion of nonalloreactive, naïve CD8+ T cells versus alloreactive central and effector memory subsets in alloMLRs. Human T cells were cultured with B-I09 or DMSO pre-treated moDCs (moDC:T cell ratio of 1:30), and additional B-I09 (20µM) or DMSO (0.1%) was added once on day 0. T cells were harvested on day +5 and surface stained for CD4, CD8, CD62L, CD45RO, and LIVE/DEAD yellow. T cells were characterized phenotypically as naïve ($CD62L^+$, $CD45RO^-$), central memory ($CD62L^+$, $CD45RO^+$), and effector memory ($CD62L^-$, $CD45RO^+$). A,B) Bar graphs show the % of each $CD8^+$ T cell subset and ratio of naïve to central + effector memory T cells in the 5-day alloMLRS (mean ± SEM). Tukey's test for A and paired t-test for B. C,D) Bar graphs show the % of each $CD4^+$ T cell subset and ratio of naïve to central + effector memory T cells in the 5-day alloMLRS (mean ± SEM). n=5 independent experiments.

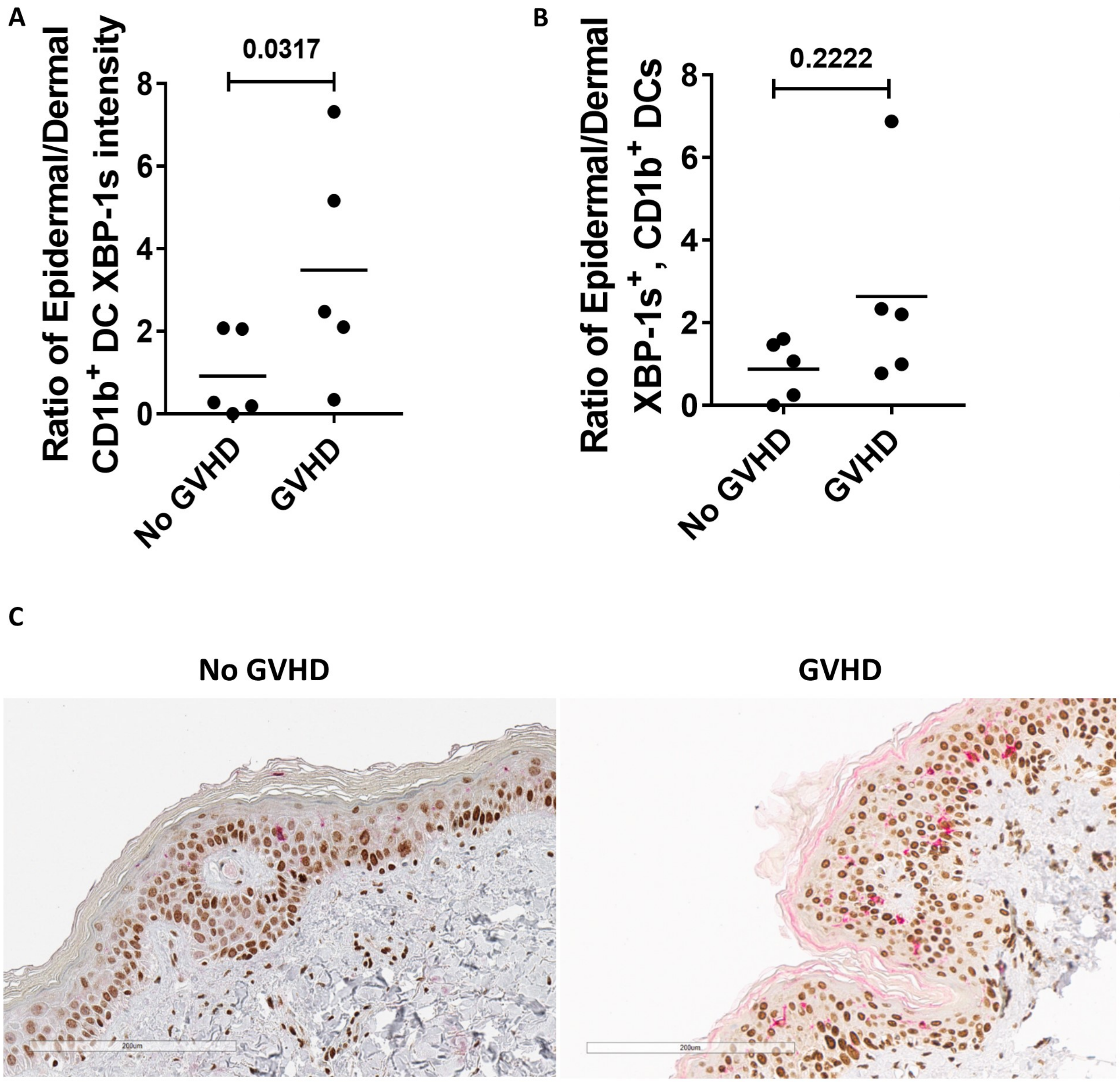

Supplemental Figure 8. XBP-1s expression in epidermal CD1b$^+$ DCs during acute GVHD. XBP-1s was evaluated in skin-resident, CD1b$^+$ DCs from a pilot group of patients (n=5 with GVHD, n=5 without GVHD). Skin biopsies were obtained from the Moffitt Cancer Center Tissue Core, consisting of tissue from consented patient. Whole slide images of skin sections were captured with an Aperio AT2 slide scan through a 20X objective lens (Leica Biosystems Inc., Buffalo Grove, Illinois). The svs image files were imported into Definiens Tissue Studio v4.7 (Definiens AG, Munich, Germany) software suite. A pathologist annotated each image into dermis and epidermis regions. Nucleus and Cytoplasm detection algorithms based on hematoxylin, DAB, and Red stain intensity and size restrictions were applied to the image to enumerate number of CD1b (DAB), XBP-1s (Red), and Dual stain positive cells. Bar graphs show the ratio of epidermal/dermal CD1b$^+$ DC XBP-1s A) staining intensity and B) amount dual positive cells from skin biopsies among patients with or without acute GVHD. Mann-Whitney test. C) Representative immunohistochemistry images are shown for each patient group, 200X.
